# Electroactive CTAB/PVDF composite film based photo-rechargeable hybrid power cell for clean energy generation and storage

**DOI:** 10.1038/s41598-022-26865-w

**Published:** 2022-12-26

**Authors:** Sanoar Molla, Farha Khatun, Ujjwal Rajak, Biswajoy Bagchi, Sukhen Das, Pradip Thakur

**Affiliations:** 1 Department of Physics, Netaji Nagar College for Women, Kolkata, 700092 India; 2 Department of Physics, Rammohon College, Kolkata, 700009 India; 3 Department of Physics, Basanti Devi College, Kolkata, 700029 India; 4 Department of Medical Physics and Biomedical Engineering, University College London, London, UK; 5 Department of Physics, Jadavpur University, Kolkata, 700032 India

**Keywords:** Energy science and technology, Engineering, Materials science, Physics

## Abstract

Herein, electroactive polymer based photo-induced hybrid power cell has been developed using CTAB/PVDF composite film in a sustainable manner. First high dielectric polymer film has been prepared by doping CTAB in PVDF matrix via solution casting method. In the basic configuration of this hybrid power cell, aqueous electrolyte solution of PVA-MnO_2_-Eosin Y has been utilized as solar light absorber and photo-electron generator whereas the high dielectric CTAB/PVDF (~ 400) is used as dielectric separator cum storage part in a very transparent way. The cell shows maximum voltage $\sim$ 1.1 V with short-circuit current density ~ 7.83 mA/cm^2^ under ~ 110 mW/cm^2^ normal light illumination. The device reveal almost same performance for a long time (30 days). The high storage impact of the hybrid cell is investigated by its promising conversion efficiency $\sim 4.48 \%$ with energy density and power density $\sim 26.9$ mWh/m^2^ and $\sim$ 5.5 W/m^2^ respectively.

## Introduction

Recently, the demand of electrical-energy conversion technologies as well as storing of the energy are effectively raising and got tremendous attention to the research world. As a result of which low-cost, highly capable and high performance self-recharging battery systems are very much demandable for the rapidly growing market of electronics devices^1^. Simultaneously, the renewable energy sources such as sunlight, wind, water, biomass energies, geothermal and etc. use is equally important and much needed for the large –scale energy production in a eco-friendly way^2^.

The performance of dye sensitized photovoltaic systems with increasing characteristics are significant part of entire global human activity^3^. These kinds of photo-sensitive cells are also a very good replacement of the natural fossil fuels (petroleum, coal etc.). Lithium-ion/sodium-ion batteries and also different types of photo-rechargeable batteries recently become very much familiar to the electronic and telecommunication field industry^4^. Although, research works on different photovoltaic devices are rapidly progressing, advanced types of polymeric nanoparticles dye-based photo-charging power unit with increasing capability of electrical energy conversion technology is still limited. So the demands of new fashioned photovoltaic devices are expanding very fast and becomes an important topic for the research field till now. Earlier, poly(vinylidene fluoride) (PVDF) based photo-rechargeable system was reported by Zhang et al. with a stored energy density of 1.4 mWh kg^−1^^5^. Ma et al., Guo et al. and Miyasaka et al. also described different types of dye based photovoltaic systems, self-charging photo-capacitors etc. previously^6,7^. High dielectric polymer thin film based inorganic–organic dye sensitive power bank type photovoltaic devices are also noted in our preceding works^8–10^.

PVDF, ([–CH_2_–CF_2_–]n) is one of the most popular polymer in research area and very much attractive to the researchers just because of its light-weight, low cost, flexible in nature, non-harmfulness. These properties are so important for the various application fields like—piezoelectric nanogenerators, capacitors, thin film transistors, grid levelling, rail runs, non-volatile memories, sensors, actuators, biomedical fields and also photovoltaic systems. α, β, γ, δ and ε are the five phases that exist in the semi-crystalline PVDF^11,12^. All trans (TTTT) planar zigzag conformation with the orthorhombic unit cell matrix makes β phase is the most electroactive phase that shows piezoelectric, pyroelectric and dielectric properties^13^. So primarily, the improvement of the $\beta$ phase content in the PVDF matrix is very essential to enhance the dielectric constant value and make it an appropriate choice for the application of photovoltaic device fabrication.

In our present work, to develop the photovoltaic energy storage device (PESD) initially, we have synthesized the cetrimonium bromide (CTAB)/PVDF composite thin film via simple solution casting method. Then the solar part i.e. MnO_2_/eosin Y has been integrated with high dielectric CTAB/PVDF composite. Here, negatively charged CTAB is chosen for the development of the dielectric property as well as the crystalline polar structure of the PVDF matrix.

CTAB is an ammonium surfactant and a good antibacterial and antifungal agent. CTAB has various applications in the field of nanoparticles synthesis (gold, silica etc.), medical and biological science and also it is a very good component for many household and cosmetics products due to its inexpensive availability, environmental stability, high adsorptive and ion exchange properties^14^. Herein, CTAB is added to pure PVDF matrix to synthesize doped PCTAB film with good dielectric value. And this high dielectric CTAB/PVDF is used with the solution of MnO_2_ NPs, eosin Y and PVA to fabricate the photovoltaic system PESD. Although ion-based battery technology is now very active in the human daily life, still there are some limitations in performance and applications with safety and low cost^15,16^. So the new kind of photo-sensitive organic dye-based devices have attracted much attention to scientists society in the recent few years.

Here, solution casting method is used to synthesize CTAB doped PVDF sample. In this process solution of 200 mg PVDF and 5 ml Dimethyl sulfoxide (DMSO) is initially prepared with 10 mass% CTAB and then it is mixed with a vigorous magnetically stirring for 12 h followed by 30 min ultra-sonication. Approximately 20 m$\upmu$ thick CTAB doped PVDF films (PCTAB10) are prepared after 6 h drying a dust-free hot air oven at 100 °C. Simultaneously, pure PVDF films with equal thickness are also prepared. The thickness of the film is measured to be ~ 20 µm. As a high dielectric insulating medium PCTAB10 is used to fabricate the PESD. 40 mg/ml PVA, 2 mg/ml eosin Y and 100 mg/ml MnO_2_ in water is stirred for 12 h at 60 °C to prepare the photo-sensitive part in the device. Then a thin layer of the aforementioned solution is casted on a conducting surface of a FTO coated glass and PCTAB10 high dielectric sample is placed on it with aluminium (0.2 cm × 0.2 cm). Here Al and FTO are acting as two electrodes for the device in which two wires are connected for the further investigation.

**Figure 1:**
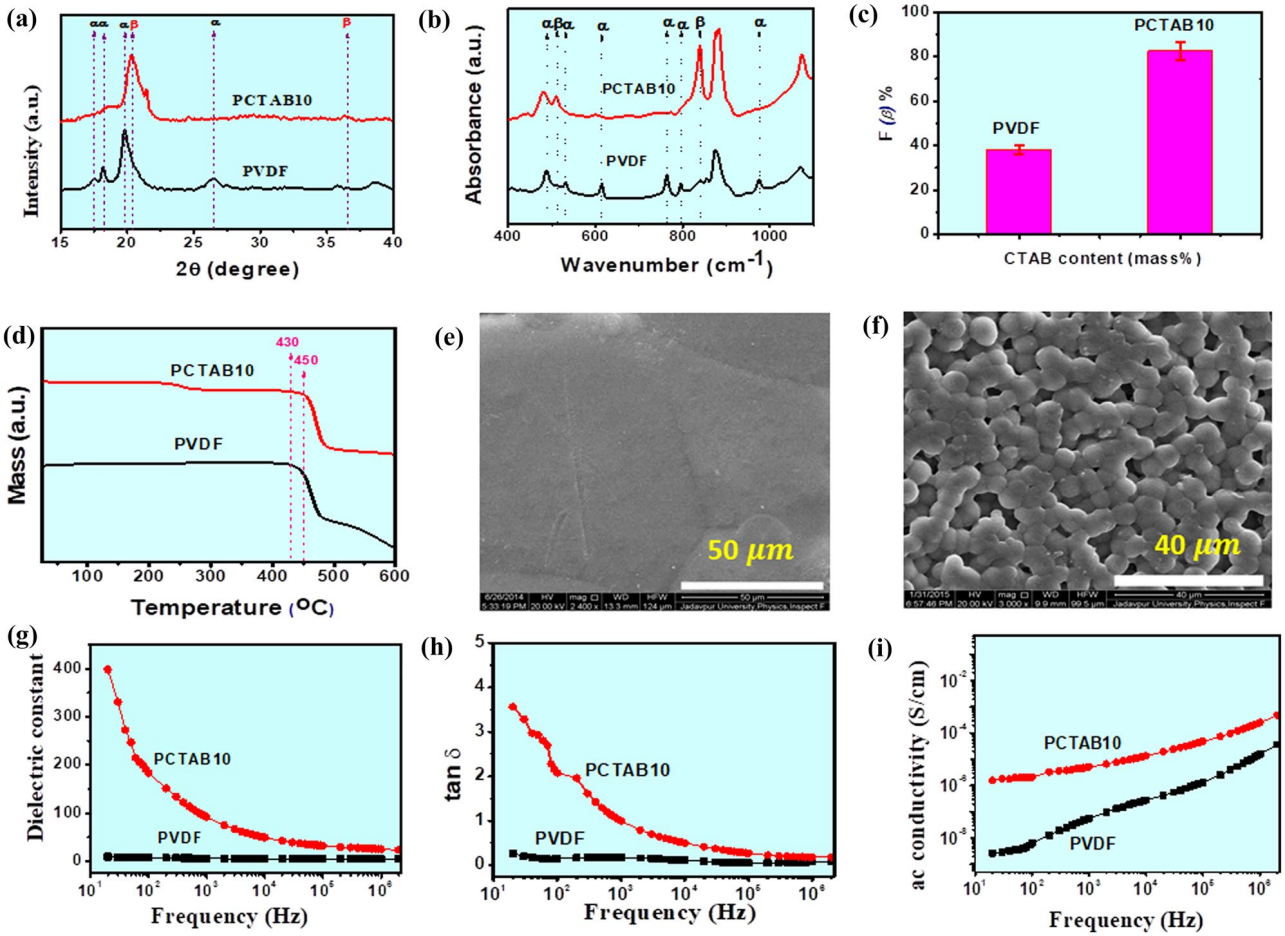
(**a**) X-ray diffraction patterns of pure PVDF and CTAB incorporated PVDF composite thin films, (**b**) FTIR spectroscopy of pure PVDF and PCTAB10 thin films (**c**) β-phase content of PCTAB10 composite sample with pure PVDF from FTIR spectra (**d**) TGA thermographs of pure PVDF and 10% CTAB modified PVDF thin films for investing the thermal stability, (**e, f**) FESEM microstructures of pure PVDF and CTAB modified PVDF films, Variation of the (**g**) dielectric constant, (**h**) ac conductivities, (**i**) tangent loss with frequency of pure PVDF and PCTAB10 composite films.

Figure 1a is the representation of the X-ray diffraction (XRD) patterns of the CTAB incorporated PVDF films and a clear indication of the electroactive β phase nucleation. This is done by the X-ray diffractometer (Model-D8, BrukerAXS Inc, Madison, WI). The diffraction peaks around 17.6° (100), 18.3° (020), 19.9° (021) and 26.6° ((201), (310)) are prominent for the existence of the non-polar $\alpha$ phase in pure PVDF. Crystalline β phase of PCTAB10 is nucleated and confirmed due to the existence of peaks at 20.8° ((110), (200))^17^. FTIR-8400S, Shimadzu instrument is used for the Fourier transform infrared spectroscopy pure PVDF and PCTAB10 films shown in Fig. 1b. $\alpha$-phase in pure PVDF ensured for the absorbance bands at 489 cm^−1^ (CF2 wagging), 533 cm^−1^ (CF2 bending), 615 and 764 cm^−1^ (CF2 bending and skeletal bending), 795 and 975 cm^−1^ (CH2 rocking), whereas, 475 cm^−1^ (CF_2_ deformation), 510 cm^−1^ (CF_2_ stretching), 600 cm^−1^ (CF_2_ wag) and 840 cm^−1^ (CH_2_ rocking, CF_2_ stretching and skeletal C–C stretching) are responsible for the crystalline $\upbeta$ phase creation in PCTAB10 sample^17^. Lambert–Beer law (equation—S1) is used for electroactive β phase is calculated and it is almost ~ 81% for PCTAB composite sample. The relative fraction of electroactive $\upbeta$ phase percentage in the concentration of CTAB10 doped PVDF films is estimated by using (equation—S1). The F($\beta$) (%) of the both samples are graphically shown in Fig. 1c. It is observed that the maximum F($\beta$) (%) is obtained $\sim 83\%$ for the doped PVDF film whereas the β-phase in pure one is just ~ 38%.

TGA thermographs are shown in Fig. 1d to study the thermal stability of pure PVDF and CTAB modified PVDF thin films. There is an only single mass loss is observed at 430 °C for pure PVDF film. However, two stage mass loss is occurred in PCTAB10 composite samples, one is ~ 220 °C and the second is 450 °C due to the decomposition of some CTAB and the PVDF respectively. The 20 °C increase in the thermo-degradation temperature in PCTAB10 samples is the confirmation of the interaction between the CTAB and the PVDF polymer matrix.^8,17^

The field emission electron microscope (FESEM) (INSPECT F50, Netherland) is used to obtain the microstructures of the pure PVDF and CTAB incorporated PVDF films which are shown in Fig. 1e,f. According to the previous study ~ 50 to 70 $\upmu$m is approximate diameter size of the spherulite of the pure PVDF but after CTAB doping the spherulite size become smaller which is the evidence of the $\upbeta$ phase formation^19^. Micrographs are also showing the uniform distribution of the CTAB particles within the PVDF polymer matrix (Fig. 1f).

Dielectric constant, tangent loss, ac conductivity—these three dielectric parameters of the pure PVDF and CTAB modified PVDF composite samples are recorded within the frequency range 20 Hz to 2 MHz by using a digital LCR meter (Agilent, E4980A). Equations S2 and S3 are used to measure the capacitance and by using this value the dielectric constant and ac conductivity are calculated. PCTAB10 composite samples have the maximum dielectric value $\sim 400$ where it is $\sim 9$ for the pure PVDF films at the lower frequency region 20 Hz (Fig. 1g). Dielectric value is consistently decreasing with increasing frequency for the PCTAB10 sample and almost constant for pure PVDF. MWS interfacial polarization can be used to explain the high value of the dielectric constant and the main reason is the proper arrangement of the large number of dipoles^18,20,21^. Figure 1h is showing the tangent loss (tan $\delta$) variation pure PVDF and PCTAB10 composite samples with frequency. From the graphical representation, the tan $\delta$ value is primarily decreasing and then it becomes constant with frequency. From Fig. 1i, no prominent change is observed in the ac conductivity value for PCTAB10 at the low frequency region but a quite linear increment is observed at the higher frequency region^18^.

So, the large dielectric (~ 400) and electroactive (~ 81%) PCTAB10 composite film is finally synthesized which is the appropriate choice to integrate the PESD as the photo charge carrier storage medium. A FTO coated glass is taken to design the PESD which contains the photo-electrons generating combined aqueous electrolyte mixture of eosin Y/MnO_2_/PVA in association with the high dielectric storage material CTAB improved PVDF film (PCTAB10) of average thickness ~ 20 m$\upmu$ and dimension 0.20 cm × 0.20 cm. As a main charge carriers suppliers eosin Y is used including with MnO_2_ and PVA which is responsible for the formation of sticky type electrolyte medium^22^. The developed PESD is charged by a normal 40 W tungsten bulb of intensity 110 mW cm^−2^ which is covered by a ultra-violet and infrared light eliminating filters.

**Figure 2:**
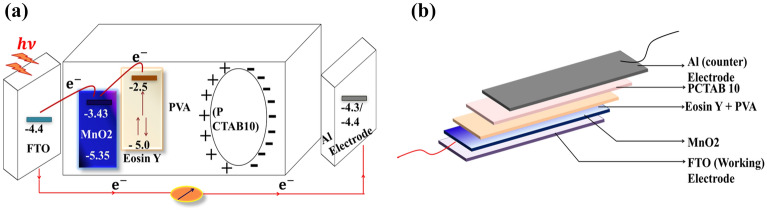
(**a**) Schematic presentation of photo charge carriers generation and storage mechanism of the PESD with HOMO and LUMO energy levels, (**b**) Schematic photograph of the PESD.

Figure 2a is the schematical representation the working mechanism of the PESD including HOMO/LUMO energy structures of the solar electrolyte with electrodes. In Fig. 2b the full structure of the PESD is shown schematically. The whole device is working with two fundamental processes i.e., photoelectrons are generated by the light sensitive part PVA/MnO_2_/eosin Y and that generated electrons are stored across the high dielectric PCTAB10 thin composite film. We have studied the photovoltaic characteristics of the PESD elaborately during both charging and discharging conditions.

**Figure 3:**
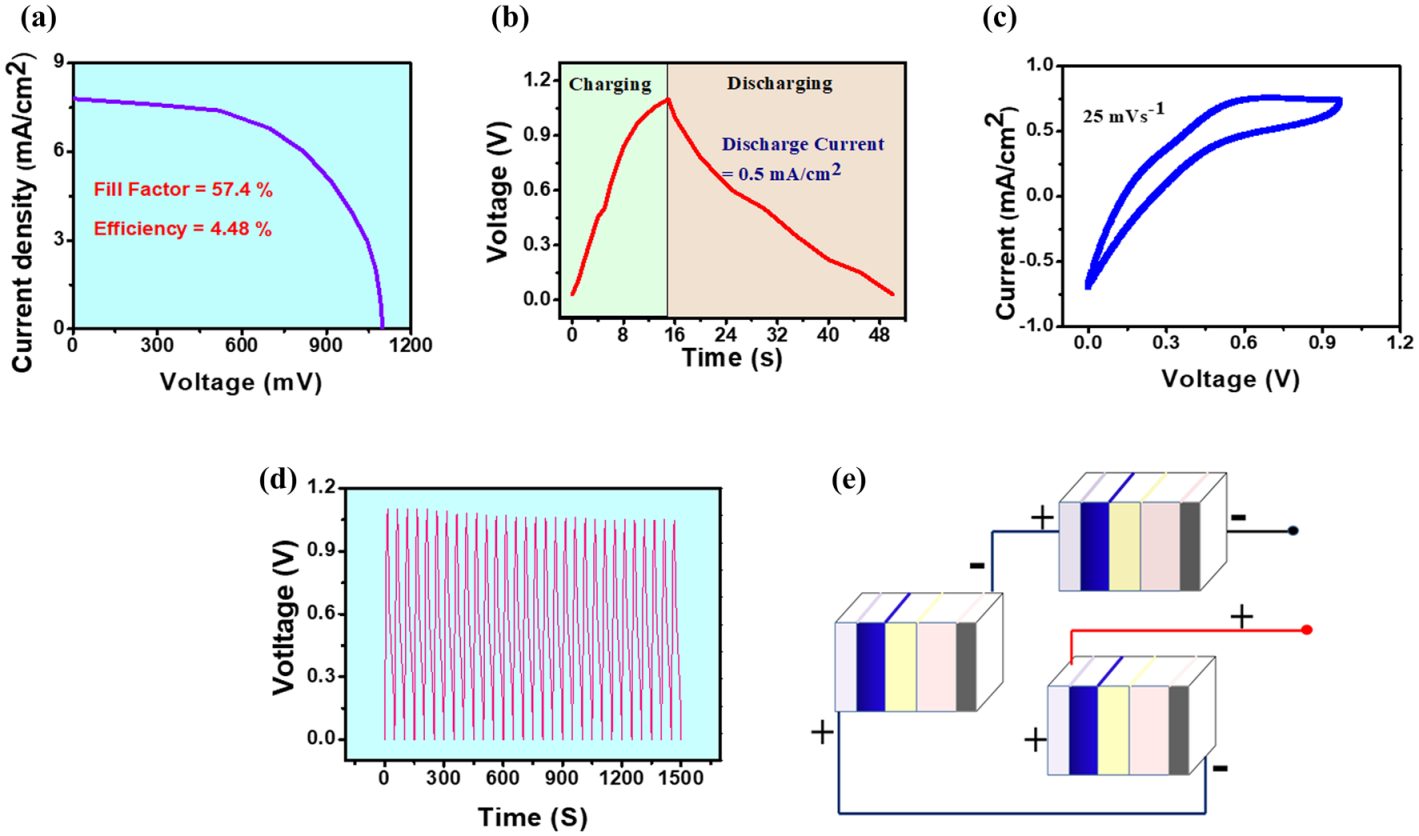
(**a**) J–V curve and (**b**) self-charging and discharging behaviour (V–t curves) of the PESD fabricated by PCTAB10 high dielectric composite thin film as a function of time under light illumination and dark conditions, (**c**) the curve of cyclic voltammetry with 0–1.2 V at 25 mV/s voltage change. (**d**) Investigation of self-charging and discharging recyclability test charge/discharge V–t profile for 30 days [1 cycle (~ 50 s) per day (N.B. 30 × 50 s = 1500 s which is shown in x-axis for the purpose of proper graphical cyclic representation)] and (**e**) self-charging (V–t) curve of three serially connected PESDs under light illumination with schematic diagram.

Graphically, current–voltage (J-V curve) and the photovoltaic behaviour of PESD are shown in Fig. 3a–c. From the variation of current density with voltage (Fig. 3a), it is observed that a very good short circuit current density (I_sc_) ~ 7.8 mA/cm^2^ is obtained for the PESD fabricated by PCTAB10. When the device is placed in front of the light illumination, at very first a large amount of charge carriers will start flowing which results the high value of short circuit current density. Figure 3b explains the self-charging and discharging phenomenon of the device. A tungsten bulb filament is used to illuminate the device so that photo-electrons of the dye eosin y can be excited to the LUMO energy state ($\sim$−2.5 eV) from the HOMO level ($\sim$−5.0 eV) by absorbing the photon particles (hν)^23,24^. After that, since the photo charge carriers are already reached to higher state it will be transmitted to lower energy state of FTO by using the LUMO level ($\sim$−3.43 eV) of MnO_2_ due to tunnelling effect^22,25,26^. Here MnO_2_ is added to the dye for making a smooth and easy tunnelling of the photo-electrons. Now, Al electrode is connected to the FTO through a conducting wire, so that the photo-electrons are rapidly start flowing to the counter electrode side. Due to the use of high insulating medium i.e. PCTAB10, photo-electrons will be reserved at the side of PCTAB10 sample and that will be acted like a negatively charged electrode. In comparison with this electrode, the conducting side of the FTO glass will be positively charged. After a while, eosin y will be unable to produce more charge carriers and then the deficit of charge carriers will be filled by the electrolyte material PVA in the aqueous solar solution.

In this project, a very simple possible mechanism is expressed to explain our device performance including the help of HOMO–LUMO energy state under solar light illumination. PVA in PVA/MnO_2_ is also acting as electron donating agent along with the dye^27^. Here our actual aim is to describe all probable electron refilling agents including the dye materials in the solar electrolyte solution. When visible light energy is fall on the device, the EY molecules and MnO_2_ nanoparticles in the photoelectrode i.e. EY/MnO_2_/PVA composite, start absorbing the photons (λ ~ 494 nm and 532 nm)^27^. The photon induced electrons of EY jump out from its HOMO (−5.0 eV) to its LUMO (−2.5 eV) energy state. Also MnO_2_ NPs are excited from its corresponding HOMO (−5.35 eV) to its LUMO (−3.43 eV) state and generate photo-electrons. Then photo-generated electrons are moved towards to the photo-electrode FTO with energy state of −4.4 eV. During photo-charging, photo-generated electrons at FTO move along the external circuit through the Cu-wire and reach the counter (Al) electrode and store in the adjoined high dielectric CTAB/PVDF (~ 400) film. The possible mechanism may be described as follows

$$\begin{aligned}& {\text{EY }} + {\text{ h}}\nu { \rightarrow } {\text{EY}}^{ + } + {\text{ e}}^{ - } , \hfill \\\,\,\, &{\text{PVA}} { \leftrightarrow } {\text{PVA}}^{ - } \hfill \\ & {\text{MnO}}_{{2}} \left( {\text{in aqueous}} \right) \, + {\text{h}}\nu { \rightarrow }[{\text{MnO}}_{{{2} - {\text{x}}}} ]^{{}} + {\text{ xe}}^{ - } \hfill \\ &{\text{MnO}}_{{2}} \left( {\text{in aqueous}} \right) \, + {\text{ PVA}} + {\text{ h}}\nu { \rightarrow } [{\text{MnO}}_{{{2} - {\text{x}}}} ]\left[ {{\text{PVA}}^{ - } } \right]^{{}} + {\text{ xe}}^{ - } \end{aligned}$$

Basically the aforementioned process can be said as the charging process of the PESD under light on condition. Since there is a potential difference between two oppositely charged electrodes as a result of which, approximately 1.1 V is obtained within 15 s (DMM 6500, Keighley). The device is charged very fast because of the rapid and smooth transportation of the electrons. From the graphical plot (Fig. 3b) the device discharged quiet slowly under light off situation within 35 s with constant current density 0.15 mA/cm^2^ (equation S4). Equation S5 is used to calculate the storage capability of PESD and that is $\sim 164$ F/m^2^ due to the superior electrochemical features of solar electrolyte regarding to charging-discharging action. The maximum output energy for PESD is attained to ~ 26.9 mWh/m^2^ with power density 5.5 W/m^2^ are calculated by using equations S6 and S7. The potentiality of the device is checked by calculating the photo-electric conversion efficiency (equations S8 and S9). To determine the storage capability of the PESD, firstly the overall efficiency of the device is estimated by using equation S10, and hence the storage efficiency of the device is found out by (equations S11). After the device performance study, all the output parameters are provided in a tabulated form in Table 1.

**Table 1:** Tabulated presentation of device performances.

Parameters	PESD
Energy density	26.9 mWh/m^2^
Power density	5.5 W/m^2^
Storage ability	164 F/m^2^
Energy conversion efficiency	4.48%
Energy storage efficiency	13%,
Overall efficiency	0.59%

All the essential equations and comparison for the device performance are supplied in the supporting information with the previously reported different types of photo-power cells (Table S1). We have done the cyclic voltammetry (CV) (PG Lyte 1.0, Kanopy Techno Solutions Pvt Ltd) action of the PESD and are shown graphically in Fig. 3c. This voltametric performance within 0–1.2 V potential range at a scan rate 25 mV/s ensures us about the good strength of electrochemical activity of the photo power cell (PESD).

The data for self-charging and discharging variation is taken for 30 days daily [1 cycle (50 s) per day] to test the constancy and staying power of the composite PCATB10 based device and there is no such notable maximum voltage drop is observed [Fig. 3d and Fig. S3 (see supporting information file)]. In Fig. 3d, we have presented the 30 cycles of data in a combined frame of common X-axis so, the range of x-axis is 0–1500 s (1 cycle ~ 50 s, so 30 cycles (observed one cycle per day for 30 days span) means 30 × 50 s = 1500 s). And maximum output voltage achieved by the devices over 30 days i.e. 30 cycles versus cycle number has also been illustrated in Fig. S3. In Fig. 3e, a schematic representation of a series connection with three PESDs is shown and that arrangement produces almost 3 V. So it is cleared that we can use our fabricated device for illuminating the commercially available different LEDs by making a series connection as a photo power bank. We have demonstrated lighting of LEDs by our fabricated cells which show its practical utilization (Video S1).

Finally, we have prepared large dielectric ( $\sim 400$) and $\upbeta$ phase nucleated CTAB doped PVDF via simple solution casting method. Then eosin Y/MnO_2_/PVA composite aqueous electrolyte solution is used to fabricate the exclusive and accessible photo-induced power cell PESD in association with the PCTAB10 composite sample as an insulating separator. Highest $\sim$ 1.1 V is attained with very good storage efficiency $\sim$ 13% including $\sim$ 5.5 mW/m^2^ power density. Furthermore, low-cost devices has been designed which may be engineered for the large scale production and to facilitate huge demands in energy sectors. And it is possible to construct a sustainable and benign nation or world by making sensible clean energy choices.

## Supplementary Information

Supplementary Information.

Supplementary Video 1.

## Data Availability

The data in support of our findings of this study are available within the paper.
