# Supplementary material for: Electroactive CTAB/PVDF composite film based photo-rechargeable hybrid power cell for clean energy generation and storage
Source: Sci Rep. 2022 Dec 26;12:22350. doi: 10.1038/s41598-022-26865-w (PMC9792523; doi:10.1038/s41598-022-26865-w)
Supplement: Supplementary file 1 — Supplementary Information. [file 41598_2022_26865_MOESM1_ESM.docx]

**Supporting Information**

**Electroactive CTAB/PVDF composite film based Photo-rechargeable Hybrid Power Cell for Clean Energy Generation and Storage**

**Sanoar Molla^1, 2^ Farha Khatun^1, 3^, Ujjwal Rajak^1^, Biswajoy Bagchi^4^, Sukhen Das^5^, Pradip Thakur*^1^**

^1^Department of Physics, Netaji Nagar College for Women, Kolkata - 700092, India.

Email address: [pradipthakurju@gmail.com](mailto:pradipthakurju@gmail.com), Mobile: +919830366215

^2^Department of Physics, Rammohon College, Kolkata-700009, India.

^3^Department of Physics, Basanti Devi College, Kolkata - 700029, India.

^4^Department of Medical Physics and Biomedical Engineering, University College London, London, United Kingdom.

^5^Department of Physics, Jadavpur University, Kolkata- 700032, India.

*Corresponding author.

**1. Materials:**

The materials that are used in our present work are poly(vinylidene fluoride) (PVDF) pellets (Aldrich, Germany . $M_{w}$: 275,000 GPC, $M_{n}$ : 71,000), poly(vinyl alcohol) (PVA) (Loba Chemie), CTAB (SRL, India), FTO coated glass (Sigma Aldrich, Germany).

**2. Equations:**

**F(**$\boldsymbol{\beta}$**) =** $\frac{\boldsymbol{A}_{\boldsymbol{\beta}}}{\left( \frac{\boldsymbol{K}_{\boldsymbol{\beta}}}{\boldsymbol{K}_{\boldsymbol{\alpha}}} \right)\boldsymbol{A}_{\boldsymbol{\alpha}}\boldsymbol{+}\boldsymbol{A}_{\boldsymbol{\beta}}}$ **(S1)**

$\boldsymbol{\varepsilon=C.d/}\boldsymbol{\varepsilon}_{\boldsymbol{0}}\boldsymbol{A}$ **(S2)**

$\boldsymbol{\sigma}_{\boldsymbol{ac}}\boldsymbol{=2}\boldsymbol{\pi f}\boldsymbol{\varepsilon}_{\boldsymbol{0}}\boldsymbol{\varepsilon tan\delta}$ **(S3)**

**Q =** ∫**I_dis_dt (S4)**

**C = Q/dV (S5)**

**E_output_ = ½ CV^2^  (S6)**

**P = VI_dis_  (S7)**

Q= the stored charge density,

C = specific areal capacitance

E_output_= output energy density

P = power density

I_dis_ = discharge current density

dt = discharge time

dV = the difference between the maximum output voltage and the voltage after complete discharge of the Device.

$\boldsymbol{ɳ}_{\mathbf{conversion}}$ **% =** $\frac{\mathbf{P}_{\mathbf{out}}}{\mathbf{P}_{\mathbf{in}}}\boldsymbol{\times}$**100 =** $\frac{\mathbf{V}_{\mathbf{OC}}\boldsymbol{\times}\mathbf{I}_{\mathbf{SC}}\boldsymbol{\times FF}}{\mathbf{P}_{\mathbf{in}}}\boldsymbol{\times}$ **100 (S8)**

V_sc_= open circuit voltage, $I_{\mathrm{SC}}$= short circuit current, FF = fill factor, $P_{\mathrm{in}}$= the incident light power (110 mW/cm^2^).

Where**, FF =** $\frac{\mathbf{V}_{\mathbf{pp}}\boldsymbol{\times}\mathbf{I}_{\mathbf{PP}}}{\mathbf{V}_{\mathbf{OC}}\boldsymbol{\times}\mathbf{I}_{\mathbf{SC}}}$ **(S9)**

$V_{\mathrm{pp}}$= voltage power point, $I_{\mathrm{PP}}$ = current power point, where $I_{\mathrm{SC}}$ and $I_{\mathrm{PP}}$ are in mA/cm^2^,$V_{\mathrm{OC}}$and $V_{\mathrm{pp}}$are in V, $P_{\mathrm{in}}$ and $P_{\mathrm{out}}$ are in mW.

Overall efficiency, $\mathbf{ɳ}_{\mathbf{overall}}\boldsymbol{\%}$ **=** $\frac{\mathbf{E}_{\mathbf{output}}}{\mathbf{E}_{\mathbf{input}}}\boldsymbol{\times}$ **100 (S10)**

Where the input energy density $E_{\mathrm{input}}$ = P_in_ $\times$d$ť$, d$ť$ is the charging time.

$\mathbf{ɳ}_{\mathbf{storage}}$**=**$\frac{\boldsymbol{ɳ}_{\mathbf{overall}}\boldsymbol{\%}}{\boldsymbol{ɳ}_{\boldsymbol{conversion \%}}}$ **(S11)**

**Figure S1:** Figure s1 shows the digital snapshot of the measurement of the thickness of the film by digital screw-gauge. It shows the thickness of the film is ~ 20 µm.


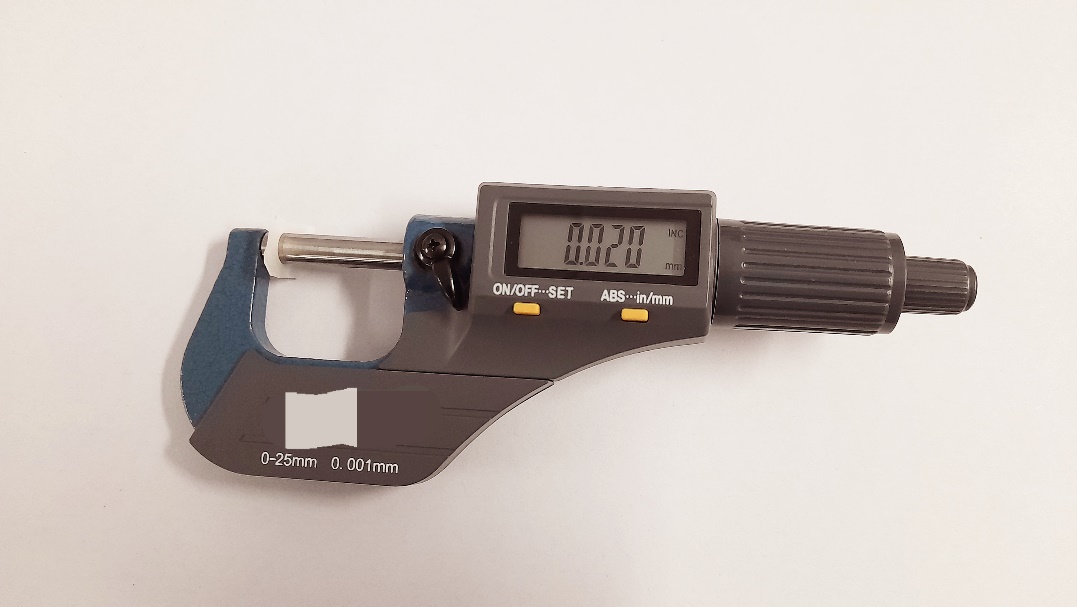


**Figure S2: T**he FESEM image of the cross-sectional image of the device part. The cross-section FESEM image shows well distribution and dispersion of CTAB in PVDF matrix and the thickness of the film is well matched with the value ~20 μm, measured by digital screw-gauge.

**
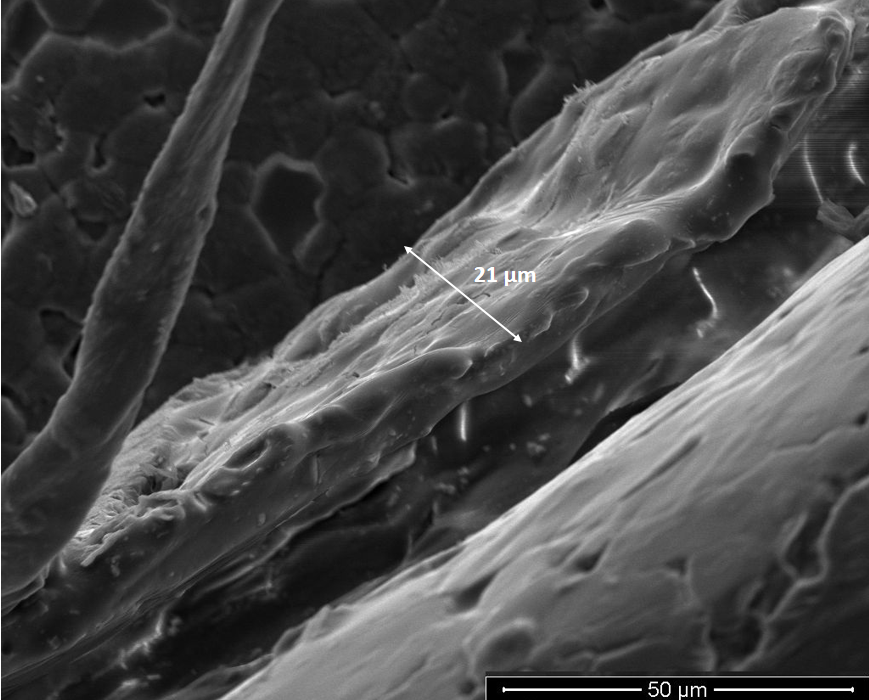
**

**Figure S3:** Maximum output voltage achieved by the devices over 30 days i.e. 30 cycles versus cycle number has also been illustrated here.





**Table – S1: Comparison of our present work with previously reported studies in tabulated form:**

| **References** | **Articles Title** | | **Energy density** | **Power density** | **Storage ability or specific capacitance** | **η_conversion_ (%)** | **η_storage_ (%)** | **η_overall_ (%)** |  |
| --- | --- | --- | --- | --- | --- | --- | --- | --- | --- |
| [1] | The photocapacitor: An efﬁcient self-charging capacitor for direct storage of solar energy | | - | - | 0.69 F/cm^2^ | - | - | - |  |
| [2] | An Integrated Power Pack of Dye-Sensitized Solar Cell and Li Battery Based on Double-Sided TiO2 Nanotube Arrays | | - | - | - | 1.95 | 42 | 0.82 |  |
| [3] | Integrated Photo-Supercapacitor Based on Bi-polar TiO 2 Nanotube Arrays with Selective One-Side Plasma-Assisted Hydrogenation | | 6.662 × 10 −8 Wh/cm^2^ | - | 1.289 mF/cm^2^ | 3.17 | 50 | 1.61 |  |
| [4] | All Silicon Electrode Photocapacitor for Integrated Energy Storage and Conversion | | 0.17 μWh/cm^2^ | 22 μWh/cm^2^ | 3.5 mF/cm^2^ | 4.8 | 43 | 2.1 |  |
| [5] | Dye-Sensitized Solar Cell with Energy Storage Function through PVDF/ZnO Nanocomposite Counter Electrode. | | 1.4 mWh kg^-1^ | - | - | 3.7 | - | - |  |
| [6] | An integrated device for both photoelectric conversion and energy storage based on free-standing and aligned carbon nanotube film. | | - | - | 54 F/g | 2.31 | 34 | 0.79 | |
| [7] | The novel “energy fiber” by coaxially integrating dye- sensitized solar cell and electrochemical capacitor. | | - | 0.27mW/cm^2^ | 3.32 mF/cm^2^ | 2.73 | 75.7 | - | |
| [8] | An ultrahigh-rate electrochemical capacitor based on solution-processed highly conductive  PEDOT:PSS films for AC line-filtering | | 1.77 mW h cm^-3^ | N.A. | 994 μF cm^-2^  Or  16.6 F cm^-3^ | - | - | - | |
| [9] | Perovskite Photovoltachromic Supercapacitorn with All-Transparent Electrodes. | | 13.4 and 24.5 mWh/m^2^ | 187.6 and 377.0 mW/m^2^ | 286.8 and 430.7 F/m^2^ | 7.8 | - | - | |
| **Present Work** | | **Electroactive CTAB/PVDF composite film based Photo-rechargeable Hybrid Power Cell for Clean Energy Generation and Storage** | **26.9 mWh/m^2^** | **5.5 W/m^2^** | **164 F/m^2^** | **4.48** | **13** | **0.59** | |
|  | |  |  |  |  |  |  |  | |

**Video S1:** Demonstration of lighting up a commercially available blue LED using our self-charged photo-power bank.

**Reference:**

^1^T. Miyasakaa, T. N. Murakami, *Appl. Phys. Lett*. 2004, **85**, 3932-3934.

^2^ W. Guo, X. Xue, S. Wang, C. Lin, Z. L. Wang, *Nano Lett.* 2012, **12**, 2520-2523.

^3^J. Xu, H. Wu, L. Lu, S.-F. Leung, D. Chen, X. Chen, Z. Fan, G. Shen, D. Li, *Adv. Funct. Mater*. 2014, **24**, 1840-1846.

^4^A. P. Cohn, W. R. Erwin, K. Share, L. Oakes, A. S. Westover, R. E. Carter, R. Bardhan, C. L. Pint, *Nano Lett.* 2015, **15**, 2727-2731.

^5^X. Zhang, X. Huang, C. Li, H. Jiang, *Adv. Mater*. 2013, **25**, 4093-4096.

^6^Z. Yang, L. Li, Y. Luo, R. He, L. Qiu, H. Lin, H. Peng, *J. Mater. Chem. A* 2013, **1**, 954-958.

^7^X. Chen, H. Sun, Z. Yang, G. Guan, Z. Zhang, L. Qiu, H. Peng, *J. Mater. Chem. A* 2014, **2**, 1897-1902.

^8^M. Zhang, Q. Q. Zhou, J. Chen, X. W. Yu, L. Huang, Y. R. Li, C. Li, G. Q. Shi, *Energy Environ. Sci.* 2016**, 9**, 2005−2010.

^9^F. Zhou, Z. Ren, Y. Zhao, X. Shen, A. Wang, Y. Y. Li, C. Surya,Y. Chai, *ACS Nano* 2016**, 10**, 5900−5908.
